# Neglected Case of Human Balantidiasis: Presumed as Antibiotic-Associated Diarrhoea

**DOI:** 10.1155/2022/6013151

**Published:** 2022-06-13

**Authors:** Sreska Shrestha, Priyatam Khadka

**Affiliations:** ^1^Sindhuli Hospital, Sindhuli, Nepal; ^2^Tri-Chandra Multiple Campus, Kathmandu, Nepal; ^3^Tribhuvan University Teaching Hospital (TUTH), Kathmandu, Nepal

## Abstract

**Background:**

Balantidiasis, due to its rare human incidence and nonspecific clinical presentations often neglected from the diagnosis considerations; however, a potent pathogen. Herein, we report a case of neglected balantidiasis presumed as antibiotic-associated diarrhoea. *Case Presentation*. A 27-year-old policeman presented in Sindhuli Hospital, with a chief complaint of epigastric pain, nausea, decreased appetite for several days, and loose stools (3-4 episodes per day). Previously, he was under antibiotic therapy (amoxycillin) for tonsillitis. The health post clinician made a presumptive diagnosis as the side effects of the antibiotics; however, the symptoms were not resolved. Complete blood cell count (CBC) was normal; renal function test (RFT) and liver function (LFT) were within the normal ranges. Ultrasonography of the abdomen and ECG (electrocardiogram) showed normal findings. However, on routine stool actively motile trophozoites of *Balantidium coli* (*B. coli*) were seen. He was treated successfully with metronidazole 750 mg tabs orally three times daily for 5 days.

**Conclusion:**

Protozoal infections, like balantidiasis, might be excluded from the diagnostic consideration in antibiotic-associated diarrhoea cases. Therefore, meticulous review of nonspecific clinical presentation and validation of an etiology with support of diagnostic tests are mandatory.

## 1. Background

*Balantidium coli*, a large pathogenic ciliated protozoan known, in rare instance infects humans resembling amoebic colitis. Despite its global distribution, even in the endemic region, the prevalence of human infection rarely exceeds 1% [1, 2]. In humans, the transmission of the ciliates occurs via the stage of the cyst which is shed on pig faeces. Therefore, humans with poor hygiene and those within the proximity to pigs are at high risk of infection—balantidiasis [2, 3]. The clinical presentation of the infection is vague, similar to that as seen in salmonellosis, shigellosis, cholera, coccidial or microsporidia, amebiasis, and other enteric infections, characterized by bloody diarrhoea or dysentery, tenesmus, nausea, vomiting, and other abdominal symptoms [2, 3]. It is, however, a potent pathogen owing to its rare human incidence and nonspecific clinical presentations often neglected from the diagnosis considerations [4–7]. Herein, we report a case of neglected balantidiasis presumed as antibiotic-associated diarrhoea.

## 2. Case Presentation

A 27-year-old policeman presented to Sindhuli Hospital with a chief complaint of epigastric pain, nausea, decreased appetite for several days, and loose stools (3-4 episodes per day). The patient had no previous history of hypertension, diabetes, TB/HIV, and other associated factors which may lead to immunosuppression. He had lived in rural areas (Baitadi district of Nepal) near a piggery farm for 5 years. As described by the patient, he was under antibiotic therapy (amoxycillin) for tonsillitis. Upon his revisit with symptoms of diarrhoea and abdominal cramps to the nearby health post the clinician thought it was a side effect of the antibiotics and suggested coming back if it worsened. The patient has kept himself hydrated with oral rehydration solution (ORS) and came to our hospital then.

## 3. Investigation

His physical examination was all well. The patient was sufficiently hydrated and the previous tonsilitis was resolved. CBC was normal; RFT and LFT were within the normal ranges. Ultrasonography of the abdomen and ECG showed normal findings. A faecal parasitological exam was requested at the Parasitology Laboratory of Sindhuli Hospital.

## 4. Physical and Microscopic Observation of Stool Sample

**Figure 1:**
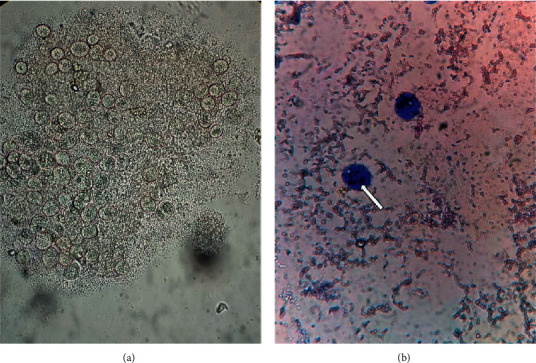
(a) Cysts and trophozoite of *Balantidium coli* (wet mount: original magnification 100×). (b) Trophozoite showing macronucleus and vacuole at the centre and cilia on outer cell wall (trichrome staining); original magnification 400×.

The received stool specimen was watery and contained blood and mucus. Immediately after receiving the sample, routine stool microscopy was performed which revealed the presence of cysts and actively motile trophozoites measuring about 50 *μ*m long and 35 *μ*m broad (Supplementary Material-1). For the better visualization of the internal structures, giemsa and trichrome staining (wheatley modification for the faecal specimen) were performed (Figure 1). Upon this morphologic characterization, the ciliated trophozoites were identified as *B. coli*.

## 5. Treatment, Outcomes, and Follow-Up

The patient was treated with metronidazole 750 mg orally three times daily for 5 days. Gradually, abdominal cramps and epigastric pain subsided within 5 days of antiprotozoal therapy. After 1 week of follow-up, we found him asymptomatic no parasites were reported on the stool sample. He was then advised to change his food habits and lifestyle.

## 6. Discussion and Conclusion

Antibiotic-associated diarrhoea is a commonly observed side effect among patients medicated with antibiotic therapy [8, 9]. In most cases, the diarrhoea is mild and requires no treatment; it clears up within a few days or after therapeutic completion [9]. Under this presumptive diagnosis, other protozoal infections might be neglected from the diagnostic consideration as had occurred in our case. Rarely occurring human infection and ability to reside asymptomatically or self-limited in some cases are the astounding characteristics of balantidiasis that possibly have baffled the diagnostic eyes.

The global distribution of balantidiasis is variable; significantly more cases are being reported from tropical and subtropical countries compared to other regions—warmer weather and high rates of rainfall provide suitable environment for parasite growth [10, 11]. Besides, poor sanitation and indigenous cultural practices are other factors behind this upset [2]. While relating balantidiasis with gender, gender should not be considered a direct risk factor because men and women have specific roles for the sustenance of their families and their roles may vary from country to country [10]. In the South Asian region, a few cases of human balantidiasis were reported [4, 6, 7, 12]. From Nepal, our case is the second known to us; the backdrop and presentation of our case compared to the previous, however, is different [12]. In the former case, the patient was characterized as having liver dysfunction due to previous antitubercular therapy (ATT); ATT has been attributed to immune suppression increasing the risk of balantidiasis. However, no such history of immune suppression was reported in our case.

Turning to our case, the policeman had no history of contact with pigs, a presumed reservoir of infection; therefore the infection might have been transmitted via food and drinks since by profession he had a mobile posting duty. The clinical presentation of the infection was mild in our cases; nevertheless, symptoms may be severe to fatal in debilitated/immunocompromised hosts—liver abscesses, peritonitis, and urogenital infection are commonly sought presentations [4–6, 13, 14]. In reference to previous findings, nutritional status, intestinal bacterial flora, parasite load, achlorhydria, alcoholism, or any chronic disease may affect the severity of the disease. However, we could not assess these parameters in our case due to the unavailability of required facilities in our hospital [1–3, 13].

Tetracyclines and metronidazole are treatments of choice for human balantidium infection. Dosage regimens and treatment durations, however, could be different depending upon the clinical manifestations and site of infection [1, 5, 13, 15, 16]. Our patient was treated with metronidazole 750 mg orally three times daily for 5 days. He responded well and recovered promptly; therefore, other antibiotics need not be prescribed. Alternatively, tetracycline 500 mg four times a day for 10 days and iodoquinol 650 mg three times a day for 20 days could be good choices [5, 14].

Among the patients medicated with antibiotic therapy, antibiotic-associated diarrhoea is common. Nevertheless, under this presumptive diagnosis, other lurking protozoal infections might be excluded from the diagnostic consideration. Therefore, meticulous review of the nonspecific clinical presentation and validation of an etiology with support of diagnostic tests is obligatory.

## Data Availability

Data generated or analyzed during this study are included in this manuscript and the remaining are available from the corresponding author on reasonable request. The video presented in the article was edited with Filmora 9 software.

## Abbreviations

ATT: Antitubercular therapy
B. coli: *Balantidium coli*
CBC: Complete blood cell count
ECG: Electrocardiogram
LFT: Liver function test
RFT: Renal function test
TUTH: Tribhuvan University Teaching Hospital.

## Supplementary Materials

Supplementary Materials Supplementary Material 1: video showing spiraling motility of *Balantidium coli* trophozoites. (video was edited with Filmora 9 software). Supplementary Material 2: informed consent in local language.
