# Supplementary material for: Neglected Case of Human Balantidiasis: Presumed as Antibiotic-Associated Diarrhoea
Source: Case Rep Infect Dis. 2022 Jun 13;2022:6013151. doi: 10.1155/2022/6013151 (PMC9208978; doi:10.1155/2022/6013151)

सुचित मन्जुरीनामा

अनुसन्धानको शीर्षक : "Neglected case of human balantidiasis: presumed as antibiotic-associated diarrhoea"

सहभागीको नाम A उमेर 26 लिङ्ग पुरुष

मलाई यस अध्ययनको बारेमा हरेक कुराको जानकारी मैले बुझ्ने भाषामा गराइएको छ। मैले सबै कुरा बुझेको छु र मलाई विशेष कुरा बुझ्ने मौका पनि दिइएको छ। मेरो सहभागिता स्वेच्छिक हो अनि कुनै पनि समयमा कुनै पनि कारण नबताई यस अध्ययनबाट बाहिरिनको लागि स्वतन्त्र छु। साथै यसले गर्दा मेरो चिकित्सकिय हेरचाहका वैधानिक अधिकार माथि कुनै प्रभाव पर्ने छैन भनी मलाई राम्ररी थाहा छ।

म आफ्ना फोटो तथा भिडियो, परिचय नखुल्ने शर्तमा दिन मन्जुर छु। मैले दिएको जानकारी मेडिकल रेकर्ड वा मेडिकल जर्नलमा प्रकाशन हुन सक्ने कुरा बुझेको छु। यस दिएको मन्जुरीनामामा मैले नै हस्ताक्षर गरेको हो।

मैले दिएका जानकारीहरु गोप्य रहने र उपचार तथा अनुसन्धानका लागि मात्र प्रयोग हुने जानकारी गराइएको छ। म आफ्नो इच्छा र स्वविवेकले नै यस अध्ययनमा सहभागी भएको छु।

यस सहमति फारममा हस्ताक्षर गर्दा म कुनै लागु औषधीको प्रभावमा नभएको कुरा स्पष्ट छु।

सहभागीको सही Arjun

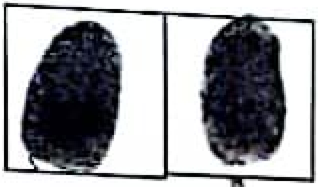

Supplement: Supplementary Materials — Supplementary Material 1: video showing spiraling motility of Balantidium coli trophozoites. (video was edited with Filmora 9 software). Supplementary Material 2: informed consent in local language. [file 6013151.f1.zip › 6013151.f1/Informed Consent in local language.pdf]
