# Supplementary material for: Neglected Case of Human Balantidiasis: Presumed as Antibiotic-Associated Diarrhoea
Source: Case Rep Infect Dis. 2022 Jun 13;2022:6013151. doi: 10.1155/2022/6013151 (PMC9208978; doi:10.1155/2022/6013151)
Supplement: Supplementary Materials — Supplementary Material 1: video showing spiraling motility of Balantidium coli trophozoites. (video was edited with Filmora 9 software). Supplementary Material 2: informed consent in local language. [file 6013151.f1.zip › 6013151.f1/Supplemental file(Video) (1).docx]

The video is available from the link: <https://drive.google.com/file/d/1AZCZNjhxy4d2e4gZwLbYHHyKzem4GENw/view?usp=sharing>
